# Abbreviated Dietary Self-monitoring for Type 2 Diabetes Management: Mixed Methods Feasibility Study

**DOI:** 10.2196/28930

**Published:** 2021-08-12

**Authors:** Kelli Marie Richardson, Gloria Cota Aguirre, Rick Weiss, Ali Cinar, Yue Liao, Kari Marano, Arianna R Bedoya, Susan Schembre

**Affiliations:** 1 Department of Nutritional Sciences College of Agriculture and Life Sciences University of Arizona Tucson, AZ United States; 2 Department of Public Health Mel and Enid Zuckerman College of Public Health University of Arizona Tucson, AZ United States; 3 Viocare Inc Princeton, NJ United States; 4 Department of Chemical and Biological Engineering Illinois Institute of Technology Chicago, IL United States; 5 Department of Kinesiology University of Texas at Arlington Arlington, TX United States; 6 Department of Family and Community Medicine College of Medicine University of Arizona Tucson, AZ United States

**Keywords:** diabetes mellitus, type 2, diet, diabetic, feasibility studies, diet records, dietary carbohydrates

## Abstract

**Background:**

Type 2 diabetes mellitus (T2D) can be managed through diet and lifestyle changes. The American Diabetes Association acknowledges that knowing what and when to eat is the most challenging aspect of diabetes management. Although current recommendations for self-monitoring of diet and glucose levels aim to improve glycemic stability among people with T2D, tracking all intake is burdensome and unsustainable. Thus, dietary self-monitoring approaches that are equally effective but are less burdensome should be explored.

**Objective:**

This study aims to examine the feasibility of an abbreviated dietary self-monitoring approach in patients with T2D, in which only carbohydrate-containing foods are recorded in a diet tracker.

**Methods:**

We used a mixed methods approach to quantitatively and qualitatively assess general and diet-related diabetes knowledge and the acceptability of reporting only carbohydrate-containing foods in 30 men and women with T2D.

**Results:**

The mean Diabetes Knowledge Test score was 83.9% (SD 14.2%). Only 20% (6/30) of participants correctly categorized 5 commonly consumed carbohydrate-containing foods and 5 noncarbohydrate-containing foods. The mean perceived difficulty of reporting only carbohydrate-containing foods was 5.3 on a 10-point scale. Approximately half of the participants (16/30, 53%) preferred to record all foods. A lack of knowledge about carbohydrate-containing foods was the primary cited barrier to acceptability (12/30, 40%).

**Conclusions:**

Abbreviated dietary self-monitoring in which only carbohydrate-containing foods are reported is likely not feasible because of limited carbohydrate-specific knowledge and a preference of most participants to report all foods. Other approaches to reduce the burden of dietary self-monitoring for people with T2D that do not rely on food-specific knowledge could be more feasible.

## Introduction

### Background

Approximately 34.2 million people in the United States have diabetes, and type 2 diabetes mellitus (T2D) constitutes 90%-95% of these cases [1]. T2D is a unique disease that can be managed through diet and lifestyle changes. Even in advanced stages of T2D that necessitate antidiabetic drugs or insulin, diet and lifestyle changes make an important contribution to glycemic stability [2]. However, according to the American Diabetes Association, knowing what and when to eat is the most challenging aspect of diabetes management [3]. Research shows that among people with T2D, who are under the impression that they are following a *diabetes diet*, only 32.6% successfully meet dietary recommendations [4]. One of the most frequently used behavior change techniques that have been shown to be effective in producing positive clinical outcomes for individuals with T2D is the *self-monitoring of behavior* [5]. Specifically, self-monitoring of diet and glucose levels can assist people with T2D to better manage their glucose levels through the improvement of multiple behavior change constructs, including goal setting, knowledge, and self-efficacy [6]. However, current self-monitoring strategies require the individual to record everything one consumes, which can be burdensome and unsustainable, and may inhibit dietary behavior change [7].

The feasibility and utility of less burdensome approaches for tracking one’s diet need to be explored to promote behavioral changes through self-monitoring. Several diet tracking approaches are currently being explored to reduce the burden of dietary self-monitoring (eg, commercial diet tracking apps and image-assisted and image-based diet tracking) [8]. However, most of these approaches require all foods to be tracked. Although this may be appropriate and expected by users interested in calorie tracking, it may not be necessary for other health promotion efforts, including the dietary self-management of T2D. As opposed to total caloric intake, the main concern for people with T2D is carbohydrate intake. When an individual without diabetes consumes carbohydrate-containing foods and beverages, the carbohydrates are broken down in the body to form glucose, and insulin is secreted by pancreatic β cells to aid the entry of glucose into the liver, muscle cells, and fat cells. In patients with T2D, the same carbohydrate breakdown process occurs, and insulin is secreted; however, the cells are resistant to insulin, causing inhibition of glucose entry into the cell, and therefore, glucose stays in the bloodstream. Due to this, a decline in insulin production occurs, and eventually, pancreatic β cells can fail, leading to a further increase in blood glucose levels. Instead of having individuals with T2D track all dietary intake, one plausible approach would be to reduce the intensity of dietary self-monitoring by tracking only carbohydrate-containing foods. This approach is consistent with historical diabetes-focused medical nutrition therapy and diabetes self-management education and support paradigms (eg, carbohydrate counting and exchange-based meal planning) promoted by the American Diabetes Association, as well as newer recommendations that encourage individualized guidance on self-monitoring of carbohydrate intake [9]. Support for the effectiveness of abbreviated dietary self-monitoring approaches comes from other areas of health promotion. A recent systematic review (Raber et al, unpublished data, 2021) showed that, even in weight loss studies where tracking all food intake would be expected, less intensive dietary self-monitoring was similarly effective as tracking all food intake. Specifically, findings showed significantly greater weight losses in the intervention groups than in the control groups in 63% of the studies where participants monitored all food intake and in 67% of the studies where participants used an abbreviated dietary self-monitoring approach (eg, tracking only certain types of foods or meals). Despite recommendations to self-monitor carbohydrate intake, there is a paucity of research examining the feasibility and effectiveness of abbreviated dietary self-monitoring approaches in the management of T2D.

### Objectives

This study aims to conduct a preliminary examination of the feasibility of a plausible abbreviated dietary self-monitoring approach for the management of T2D, in which only carbohydrate-containing foods are recorded in a diet tracker. We hypothesized that this less intensive dietary self-monitoring approach would be feasible if people with T2D (1) have general diabetes knowledge and diabetes-related nutrition knowledge and (2) find this approach acceptable based on the ease of use and a preference over recording all foods.

## Methods

### Study Design

**Figure 1:**
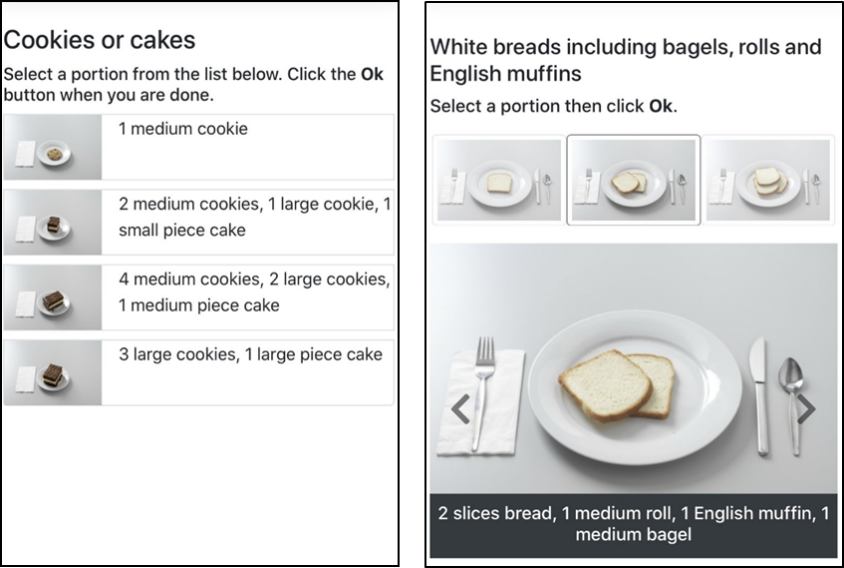
Portion size estimation methods: portion list (left) and carousel (right).

This study is a secondary analysis of data collected in phase I of the project mDGS (mobile dietary guidance system). Project mDGS aims to develop an mDGS that assists people with diabetes self-management. The objective of phase I of project mDGS is to evaluate the usability of three functional prototypes of the food entry interface and two functional prototypes of the portion size estimation interface designed for the mDGS mobile app. The methods for food entry were selected from previously validated mobile-based research tools [6,10,11]. These methods include the following: (1) text entry, where the user enters a food description to search for a specific food; (2) tree structure, where the user works through a hierarchical tree structure based on food groups and subgroups (ie, grains-bread-whole grain) to find and select a specific food; and (3) food group, where the user is directed through a food list by designated food groups as performed by food frequency questionnaires. The methods for portion size selection depicted in Figure 1 include a portion list and a carousel [11]. A full set of smaller food portion images is displayed using the *portion list* method. The user then selects one picture to expand for better viewing. The *carousel* type of portion size estimation displays images for the user to select from by swiping left or right. Other options for diet tracking have been developed, including voice-based searching or barcode scanning; however, these methods were not tested because their utility is limited or not well validated in research. The primary results from the evaluation were used to inform the final design specifications for the mDGS app. Here, we describe the secondary findings of the evaluation. Specifically, this secondary analysis examines the qualitative and quantitative data collected to assess the feasibility of using mDGS to record the intake of only carbohydrate-containing foods compared with that of all foods. Project mDGS was reviewed and approved by the University of Arizona Human Subjects Protection Program. All enrolled participants provided informed consent in either written or electronic form.

### Study Recruitment

Participants were recruited using in-person (pre–COVID-19 pandemic) and remote (during the pandemic) methods. The in-person recruitment methods included flyers placed in a primary care clinic, attendance at a community health fair, and an information table staffed by research personnel in the lobby of the university-affiliated diabetes clinic. The in-person recruitment method yielded 4 enrolled participants. In March 2020, all recruitment efforts were conducted remotely using ResearchMatch following institutional guidance to cease in-person research. A total of 487 emails were sent to ResearchMatch volunteers with T2D, with 57 people indicating interest in the study. One additional participant was recruited via a standing contact form on the department website, and another participant was recruited via word of mouth. Of the 59 interested people, 18 (31%) did not respond to further contact attempts, and 5 (8%) did not attend their scheduled data collection sessions. Furthermore, 5% (3/59) of interested people declined because of time constraints (n=1), security concerns regarding the virtual platform (n=1), and mention of needing a social security number for tax reporting purposes related to participant compensation in the consent form (n=1). One potential participant was not enrolled because the recruitment goal was already met.

Interested participants were screened using an eligibility questionnaire. Eligible individuals were those aged at least 18 years, had been diagnosed with T2D for at least 6 months, were fluent in English, familiar with the use and functionality of mobile apps (ie, using a mobile app at least once per week), and willing to use a health-related mobile app in the future. Exclusion criteria were unwillingness to use a mobile app for T2D management, inability to attend an in-person or virtual data collection session, and the use of a mobile app less than once a week. Statistics on comorbid conditions were not collected for this study. One interested person was excluded because of a lack of mobile app use. In total, 31 participants enrolled and participated in the data collection interview; however, 1 participant experienced technical difficulties during the data collection process, resulting in substantial missing data. This participant was excluded from the analyses, resulting in an analytical sample of N=30.

### Data Collection Procedures

Eligible individuals were scheduled for 1-hour interview sessions. For in-person meetings, the sessions began with informed consent procedures and the completion of quantitative surveys on demographics, medication use, mobile technology use, personal experience with diabetes, and general diabetes knowledge. Remote data collection sessions were similar, except that participants provided consent and completed questionnaires on REDCap (Research Electronic Data Capture), and interview sessions were conducted on the Health Insurance Portability and Accountability Act–compliant Zoom for Health platform (Zoom Video Communications). The study staff obtained permission to audio record the interviews. All interview sessions consisted of guiding participants to access a functional version of the web-based mDGS mobile app and to trial each of the three prototyped food entry interfaces and the two portion size interfaces by entering sample meals (breakfast, lunch, and dinner) consisting of typically consumed foods in standard US portion sizes into the app. All meals included foods that were high and low in glycemic index and had a moderate-to-high glycemic load (glycemic load>15). The order in which participants tested the food entry and portion size selection interfaces was randomized to omit the effect of order on the evaluation. The research staff asked evaluation questions designed to assess the acceptance and utility of each interface. In addition, participants were asked, using qualitative and quantitative methods, for their opinions on the mDGS concept and their thoughts on the future directions for the app. As the parent study was focused on the functional usability of the mDGS diet tracker, no additional context for using the app was provided (ie, time frame for recording intake). For this study, only data specific to questions on general diabetes knowledge and diabetes-related nutrition knowledge and the acceptability of reporting only carbohydrate-containing foods versus all foods were analyzed. The related measures are described in the following sections.

### Measures of Diabetes Knowledge

General diabetes knowledge and diabetes-related nutrition knowledge were assessed using the brief Diabetes Knowledge Test developed by the Michigan Diabetes Research Training Center [12]. Of the 23 knowledge test items, only the first 14 (61%) items, which were nonspecific to insulin use, were included. The general test component’s reliability is demonstrated by a coefficient α of .77, and its face validity is supported by the consistency observed in four separate analyses [12]. Diabetes-related nutrition knowledge was computed from the 5 items specific to nutrition in a manner similar to that of a previous study [13]. The Diabetes Knowledge Test was not completed by the first 4 study participants, as it was added to the study when the protocols were modified for remote data collection.

Diabetes-related nutrition knowledge specific to identifying carbohydrate-containing foods was additionally assessed using a study-specific task similar to that used in previous research [14]. Participants were asked to categorize 10 commonly consumed food items as containing or not containing carbohydrates (≤5 g). A random-ordered list of 5 carbohydrate-containing and 5 noncarbohydrate-containing foods was provided to all participants. The 5 carbohydrate-containing foods were coffee with cream and sugar, a turkey sandwich, strawberries, hash browns, and orange juice. The 5 noncarbohydrate-containing foods were steak, bacon, eggs, unsweetened green tea, and steamed broccoli. Participants were instructed to check all food items that they considered to contain carbohydrates.

### Measures of Acceptability

As part of the interview, acceptability was assessed quantitatively on a 10-point Likert scale as the perceived difficulty of reporting only carbohydrate-containing foods, and their preference for reporting only carbohydrate-containing foods versus all foods was recorded qualitatively. Participants were also asked to provide reasons for their answers. Preference for reporting dietary intake was assessed by the following question: “Would you be interested in using a diet tracker that focused only on foods and beverages that interfere with good diabetes management vs. a diet tracker that requires you to enter ALL the foods and beverages you eat?” All interviews were recorded and transcribed. Furthermore, 2 trained research staff independently coded the transcribed interviews by identifying the themes in participant responses. A third researcher reviewed the coding and resolved any discrepancies before the analysis. Quantitative reports of acceptability were completed by averaging the reported scores on a 10-point Likert scale. Qualitative analyses were completed manually by quantifying the number of participants who stated they would prefer to enter all foods, just carbohydrate-containing foods, or had mixed opinions based on the question presented above. The label *mixed opinions* was provided to individuals if they preferred to start tracking one way and then switch to the other or if they wanted both options to be available. The reasons for perceived difficulty were categorized based on similar responses and quantified.

### Statistical Analysis

Descriptive statistics were used to characterize study participants. Tabulated and qualitative data are presented as frequencies. Quantitative data are summarized as means, SDs, and ranges.

## Results

### Sample Characteristics

In total, 30 participants completed the study. The participants were predominantly female (18/30, 60%), non-Hispanic (27/30, 90%), and White (25/30, 83%). They ranged in age from 28 to 78 years with a mean age of 58.6 years (SD 11.9) and represented each of the 5 regions of the United States. Most reported having T2D for 6-10 years (12/30, 40%), followed by 0.5-5 years (7/30, 23%), and 11-15 years (7/30, 23%). Nearly all participants (27/30, 90%) reported having had prior T2D education (Table 1).

**Table 1:** Participant characteristics and Diabetes Knowledge Test scores (N=30).

Variable		Statistic
Age (years), mean (SD)		58.6 (12.1)
Sex (female), n (%)		18 (60)
Ethnicity (non-Hispanic), n (%)		27 (90)
**Race, n (%)**		
	White	25 (83)
	Black or African American	3 (10)
	Pacific Islander	1 (3)
	Declined to answer	1 (3)
**Years with T2D^a^, n (%)**		
	0.5-5	7 (23)
	6-10	12 (40)
	11-15	7 (23)
	16-20	2 (7)
	>20	2 (7)
Prior T2D education (yes), n (%)		27 (90)
**Diabetes Knowledge Test^b^**		
	Test score, mean (SD)	83.4 (14.2)
	Participants with test score>65%, n (%)	22 (88)
Diabetes-related nutrition knowledge score^b,c^, mean (SD)		82.7 (20.1)

^a^T2D: type 2 diabetes.

^b^Five participants did not complete the Diabetes Knowledge Test.

^c^Diabetes-related nutrition knowledge was computed from the items on the Diabetes Knowledge Test specific to nutrition (ie, items 1, 2, 3, 4, 7, and 12).

### Measures of Diabetes Knowledge

The mean score of the participants in the Diabetes Knowledge Test was 83.9% (SD 14.2%; range 16.7%-100%), and the mean score of the diet-related Diabetes Knowledge Test questions was 82.7% (SD 20.1%; range 16.7%-100%). The median score of the carbohydrate-containing foods knowledge task was 80% (range 40%-100%), reflecting an average of 8 correctly categorized foods. Only 20% (6/30) participants correctly categorized all 10 foods. The carbohydrate-containing foods most incorrectly classified by participants were strawberries (11/30, 37%), coffee with cream and sugar (10/30, 30%), orange juice (10/30, 30%), and steamed broccoli (7/30, 23%).

### Measures of Acceptability

The first measure of acceptability of recording only carbohydrate-containing foods versus all foods with the mDGS app was *perceived difficulty*. In the quantitative analysis, the mean perceived difficulty of recording only carbohydrate-containing foods was 5.3 (SD 2.5; range 1-10; 1=not difficult at all and 10=very difficult). From the qualitative analysis, the most cited reasons for greater perceived difficulty were not knowing what foods contain carbohydrates (20/30, 67%), the acknowledgment that certain foods may vary in carbohydrate content by brand (3/30, 10%), and the acknowledgment that not all carbohydrates are *bad* (2/30, 7%). One participant who reported not knowing what foods contain carbohydrates stated as follows:

...sometimes you think that certain things do not contain carbs because when you think of carbs, you think of (at least I do) of bread, pasta, rice, beans. You think of those things as carbs, so there might be other things that contain carbs that I don’t know right away, so I may say that just reporting things [containing carbs] would be difficult because I don’t know exactly what does not contain carbs.

Another participant, who reported not knowing what foods contain carbohydrates, stated the following:

I’m not sure which has carbs, and which don’t sometimes. Isn’t practically every food have a little bit of carb or something? I don’t know.

Regarding the variability in carbohydrate content of certain foods, one participant stated the following:

Like a sugar free candy is fine with carbs, but umm you can find some sugar free sweet of some kind, but it doesn’t mean that it is umm, it will say ‘doesn’t impact sugar’ like these net carb things like Atkins sweets. But it doesn’t mean that, and it’ll say, ‘does not contain sugar or will not impact carbs, does not impact calories’ and so you have to report it typically on these kinds of apps, and that’s the tedium.

Regarding the relative quality of some carbohydrate-containing foods, another participant stated the following:

Well, some are very obvious, but others like the strawberries or the broccoli, you know they have some carbs, but we don’t necessarily know. I mean, most people probably wouldn’t think of strawberries having carbs.

A second qualitative measure of acceptability was the reported preference for recording carbohydrate-containing foods only or all foods. Approximately half (16/30, 53%) of the participants reported a preference for recording all foods over recording carbohydrate-containing foods only, 30% (9/30) reported a preference for recording carbohydrate-containing foods only, and 17% (5/30) wanted to have both options. The top-cited reasons for preferring to record all foods over only carbohydrate-containing foods were being unknowledgeable about what foods contain carbohydrates (12/30, 40%) and wanting additional dietary feedback related to diabetes management (8/30, 27%; eg, calorie tracking for weight loss). Regarding the lack of knowledge about what foods contain carbohydrates, one participant stated the following:

I mean I think it would feel easy, but I’d probably be wrong. Before I did this little exercise today, I would’ve probably labeled bacon as a carb because it can be fatty...I confuse fat with carbs.

Another participant wanted to record all foods because it was easier not to have to think about which foods were carbohydrate-containing and stated:

Probably it would be all the foods because you know, you do things sometimes automatically and don’t think about it.

A participant who was interested in receiving additional dietary feedback on the total caloric intake stated as follows:

I need to enter everything in there...so I can know where I’m at you know. It’s like, okay, they tell you you can have so many calories a day. Well, if I overused all of them, I want to know. I need to have something telling me that, and it’s only going to happen if I enter everything in.

Another participant interested in receiving additional dietary feedback, who was less confident about his diabetes-related nutrition knowledge, stated the following:

I want to know how much protein I’m eating and how much fat I’m eating too. But that could be my ignorance about diabetes. I haven’t educated myself nor has a dietitian given me the overview picture of what every food group does to my blood sugar. So, I just think I need to enter all foods.

Later, this participant said he wanted to report all foods because he was also focused on weight loss:

My point was I had to lose weight, so I was being coached in being a diabetic within the context of having to lose weight.

All 9 participants who reported a preference for recording carbohydrate-containing foods only versus all foods cited that recording carbohydrate-containing foods only would reduce the burden associated with reporting all foods. One participant stated as follows:

If you report everything...that is where the tedium comes in, where you’re trying to count every little, tiny, nit-picky thing.

Another participant, when offered the option of recording carbohydrate-containing foods only, stated the following:

...that would make my life a lot easier. That is with managing diabetes, not necessarily for weight loss. But for managing diabetes, yes.

Finally, among those who wanted both options (5/30, 17%), 4 participants noted the educational benefits of first recording all foods for some time until they were confident in their ability to record only carbohydrate-containing foods, with one participant stating:

I can probably do the one with everything first and learn a little more and then if I felt confident and, like, I was ready to do the just the one.

Another participant, who first wanted to start by recording all foods and then move to recording carbohydrate-containing foods only, specifically noted the burden of having to record all foods:

...Recording everything could be too cumbersome. But if you could figure out what is helping you versus what isn’t helping you that way, you know what I mean, [mDGS could] be a program you can use. The problem is when you get too involved with trying to put too many details in your diet and trying to watch everything it becomes overwhelming.

The last participant with a mixed opinion thought that having the option to enter all foods or only carbohydrate-containing foods would be beneficial to users who may have goals in addition to diabetes management. This participant stated as follows:

I think the app should be set up in such a way that you can have multiple objectives. And if your objective is to monitor your diet and learn to eat better portion sizes and stuff like that, then all foods. If you’re just worried about the diabetes type of questions, then just the carb-containing foods. But I think that should be something that somebody can decide for themselves. Those options can easily be in the same app.

## Discussion

### Principal Findings

This study aimed to examine the feasibility of an abbreviated dietary self-monitoring approach in which only carbohydrate-containing foods are recorded in a mobile diet tracker. We hypothesized that this approach would be feasible if people with T2D (1) had diabetes-related nutrition knowledge to report only carbohydrate-containing foods and (2) found the approach acceptable based on the ease of use and a preference for this approach over recording all foods. However, our results did not support the feasibility of this specific approach. First, we found that diabetes-related nutrition knowledge was highly variable among participants, with proportionally few participants being able to classify 10 commonly consumed foods as containing versus not containing carbohydrates (≤5 grams). Second, we found that approximately half of the participants reported a preference for recording all foods versus carbohydrate-containing foods, with most participants citing a lack of knowledge about carbohydrate-containing foods as the primary barrier to acceptability.

Despite observing seemingly adequate mean levels of general diabetes knowledge and diabetes-related nutrition knowledge in this study, we observed an insufficient knowledge base on carbohydrate-containing foods that would be necessary for the proposed abbreviated dietary self-monitoring approach. Participants in this study had relatively good general diabetes knowledge compared with other studies of people diagnosed with T2D for a similar duration. Hashim et al [15] reported a mean Diabetes Knowledge Test score of 55%, and Almalki et al [16], who also used the Diabetes Knowledge Test, showed that only 21.6% of participants had good diabetes knowledge (defined as scoring >65%). However, in the studies by Hashim et al [15] and Almalki et al [16], it is unclear whether participants had received diabetes education, which may explain the higher mean diabetes knowledge scores observed in this study, where 90% (27/30) of participants reported having had diabetes education. Among a similarly educated sample of people with T2D, Breen et al [13] reported that diabetes-related nutrition knowledge was modest based on average scores of 60% on the diet subsection of the Audit of Diabetes Knowledge Questionnaire. These results are consistent with our findings and further support that diabetes-related nutrition knowledge is suboptimal, even among those who have had diabetes education.

Another important finding was that, regardless of diabetes-related nutrition knowledge, half (14/30, 47%) of the study sample expressed a preference for reporting carbohydrate-containing foods only or moving between reporting carbohydrate-containing foods and all foods, based on their health behavior goals (eg, glucose stability or weight loss) or as they gained confidence in identifying carbohydrate-containing foods. With regard to diet tracking for weight loss, there may be expectations that tracking total caloric intake is necessary for successful weight loss despite previously reviewed evidence (Raber et al, unpublished data, 2021, [17]) reporting that abbreviated dietary self-monitoring and other behavioral weight loss strategies (eg, daily self-weighing) are similarly, if not more, effective. On the basis of this supporting literature and the findings of this study, the feasibility and efficacy of other abbreviated dietary self-monitoring approaches should be explored. One plausible approach, which would minimize the reliance on diabetes-related nutrition knowledge, is to provide a (personalized) list of commonly consumed carbohydrate-containing foods and have users select only those that they have consumed, an approach similar to ecological momentary diet assessment approaches [8].

### Strengths and Limitations

A strength of this study is that we used both quantitative and qualitative data collection approaches to obtain enriched data on the feasibility of the proposed, abbreviated dietary self-monitoring approach. In addition, recruitment was remote, which led to a geographically diverse sample and the ability to better generalize our results. Finally, participants were provided context for reporting dietary intake in the parent study (eg, evaluating mobile diet tracker prototypes), which might have enhanced their consideration of the stated options for reporting. One of the limitations of this study was that this was a secondary analysis of data collected as part of the evaluation of a mobile diet tracker prototype; therefore, methods were not designed to assess the efficacy of abbreviated dietary self-monitoring. In addition, the sample was small and predominantly White; however, those enrolled were almost equally represented men and women and were diverse in terms of age and duration of diabetes diagnosis. The sample only consisted of participants who were familiar with the use and functionality of mobile apps in general, which may have skewed preference for use; however, this sample was selected to reflect those who may actually consider using an app for diet tracking. Food insecurity and other social determinants of health were not assessed, both of which could affect general diabetes and diabetes-related nutrition knowledge; however, 90% (27/30) of participants had received diabetes education, which implies that our participants had adequate access to resources. Future work to develop the mDGS app will need to address this limitation by assessing participants for food insecurity or other social determinants of health before study participation and data extraction to determine if this has an impact on study results. Although we only asked a few questions regarding diabetes-related nutrition knowledge, to our knowledge, this is one of the few studies that used a simple but effective carbohydrate-containing food knowledge task to assess knowledge about carbohydrate-containing foods. Finally, our sample performed comparatively better on the Diabetes Knowledge Test than the populations sampled in the previously cited studies, which could have biased our findings. However, the inclusion of the carbohydrate-containing foods knowledge task, which highlighted a discrepancy between general diabetes knowledge and diabetes-related nutrition knowledge in our population, and the inclusion of qualitative and quantitative measures of food reporting preference strengthened our conclusions.

### Conclusions

Our findings suggest that this abbreviated dietary self-monitoring approach may not be feasible, particularly for those with limited knowledge of carbohydrate-containing foods. Despite these findings, this study adds to the paucity of literature that explores options for less intensive dietary self-monitoring for the management of T2D. On the basis of a review (Raber et al, unpublished data, 2021) supporting the efficacy of less intensive dietary self-monitoring in other areas of health promotion, these findings do not rule out the potential efficacy of abbreviated dietary self-monitoring approaches for T2D management altogether. It is important to acknowledge that nearly half of the participants in this study, regardless of their diabetes-related nutrition knowledge, reacted positively to only having to record carbohydrate-containing foods, which is consistent with published reports [4] on the burden of dietary self-monitoring. Furthermore, removing the barrier of limited diabetes knowledge could significantly shift the preference for and feasibility of abbreviated dietary self-monitoring for the management of diabetes. Collectively, these findings suggest that offering users a choice to record all foods versus record only carbohydrate-containing food or identifying other abbreviated dietary self-management approaches that rely less on diabetes-related nutrition knowledge could be an effective diabetes self-management strategy.

## Abbreviations

mDGS: mobile dietary guidance system
REDCap: Research Electronic Data Capture
T2D: type 2 diabetes mellitus
